# Relationship between Dynamics of TNF-α and Its Soluble Receptors in Saliva and Periodontal Health State

**DOI:** 10.3390/dj10020025

**Published:** 2022-02-08

**Authors:** Ryota Kibune, Kosuke Muraoka, Masaki Morishita, Wataru Ariyoshi, Shuji Awano

**Affiliations:** 1 Department of Clinical Education Development and Research, School of Dentistry, Kyushu Dental University, 2-6-1 Manazuru, Kokurakita-ku, Kitakyushu 803-8580, Japan; r17kibune@fa.kyu-dent.ac.jp (R.K.); muraoka@kyu-dent.ac.jp (K.M.); r08morishita@fa.kyu-dent.ac.jp (M.M.); 2 Department of Infections and Molecular Biology, School of Dentistry, Kyushu Dental University, 2-6-1 Manazuru, Kokurakita-ku, Kitakyushu 803-8580, Japan; r11ariyoshi@fa.kyu-dent.ac.jp

**Keywords:** periodontitis, saliva, tumor necrosis factor α, soluble TNF receptors

## Abstract

Soluble tumor necrosis factor receptors 1 and 2 (sTNF-R1 and sTNF-R2) are reported to protect against excessive TNF-α, a primary mediator of systemic responses to infection. This study aimed to investigate the levels of TNF-α, sTNF-R1, and sTNF-R2 in saliva and to verify whether their dynamics are associated with periodontal health. The study population comprised 28 adult patients. Probing pocket depth, clinical attachment level, and bleeding on probing were assessed, and periodontal inflamed surface area (PISA) was calculated. Stimulated saliva was collected before the oral examinations. The levels of TNF-α, sTNF-R1, sTNF-R2, and total protein (TP) in saliva samples were determined. There were significant positive correlations between TNF-α, sTNF-R1, and sTNF-R2 to TP (/TP) in stimulated saliva. Moreover, there were significant positive correlations between PISA and sTNF-R2/TP. Stepwise multiple regression analysis revealed that PISA was significantly associated with sTNF-R2/TP in saliva; however, TNF-α/TP was not significantly associated with PISA. In conclusion, this study demonstrates that significant relationships exist between the salivary levels of TNF-α and sTNF-R1, and that salivary sTNF-R2 is associated with the expansion of inflamed periodontal tissue.

## 1. Introduction

Tumor necrosis factor-α (TNF-α) is a major mediator of inflammation and inflammation-related diseases [1]. It acts as a pro-inflammatory cytokine that plays a central role in immune regulation and a variety of inflammatory responses during destructive periodontal disease [2].

The effects of TNF-α are mediated by two membrane receptors, TNF receptor type 1 (TNF-R1) and TNF receptor type 2 (TNF-R2), that are carried on the surface of the target cell [3]. These two receptors bind TNF-α with high affinity; however, they have different localizations and physiological effects [4]. TNF-R1 is expressed in a variety of cells, and its overexpression is involved in the induction and exacerbation of inflammatory responses, whereas TNF-R2 is expressed in a limited number of cells, such as endothelial, epithelial, immune, and fibroblasts, and is involved in disease remission [5,6].

A disintegrin and metalloproteinase 17 (ADAM17) is a TNF-α-converting enzyme, which is the principal protease involved in the activation of pro-TNF-α and is assumed to be the main protease responsible for the release of TNF-α from the transmembrane [7]. We previously reported that the severity of periodontal disease may be associated with the expression of the ADAM17 gene in the human buccal mucosal epithelium [8], and that ADAM17 is strongly expressed in the epithelium of gingival tissues and regulates the production of TNF-α from oral keratinocytes [9]. Furthermore, TNF-R1 and -R2 are cleaved and separated from the cell membrane region by ADAM17 and converted into soluble TNF receptor type 1 (sTNF-R1) and soluble TNF receptor type 2 (sTNF-R2), respectively [7,10]. sTNF-R competes with transmembrane TNF-R and inhibits the binding of TNF-α to transmembrane TNF-R, thereby inhibiting the action of TNF-α [10,11,12]. TNF-α is involved in the development and exacerbation of autoimmune and inflammatory diseases such as rheumatoid arthritis [13]. sTNF-R has already been clinically applied as a specific medicine to regulate the action of TNF-α during the inflammatory response in rheumatoid arthritis [14]. However, it remains unclear how sTNF-R is related to inflammatory oral diseases, such as periodontitis, in which TNF-α is involved in the development of inflammation. Previous studies have reported that the stimulation of TNF-α induces sTNF-R2 shedding from gingival fibroblasts in vitro [15], and that TNF-α, sTNF-R1, and sTNF-R2 in gingival crevicular fluid (GCF) are significantly increased in chronic periodontitis [16].

Whole saliva is a fluid containing components of the exocrine glands and GCF in the oral cavity and is considered a diagnostic fluid for periodontal disease screening. In fact, various mediators of chronic inflammation and tissue destruction have been detected in the saliva of patients with periodontitis and are thought to reflect the state of oral health, including periodontal disease status [17], and it has been reported that the salivary levels of TNF-α are elevated in patients with clinical indicators of periodontitis [18]. Thus, this study investigated the levels of TNF-α, sTNF-R1, and sTNF-R2 in the saliva and verified whether their dynamics are associated with periodontal health.

## 2. Materials and Methods

### 2.1. Participants

The study population comprised 28 adult patients (11 men and 17 women), randomly selected from patients with slight or moderate chronic periodontitis, who visited the Kyushu Dental University Hospital from December 2019 to November 2020. Their average age was 67.2 (standard deviation (SD): 15.8) years, and the average number of teeth was 25.2 (3.4). Patients with medical disorders, those who had taken antibiotics or other antimicrobial therapy within the past 3 months, those who had fewer than 20 teeth, and pregnant women were excluded from the study.

The human subject protocol was approved by the Ethics Committee of the Faculty of Dentistry at Kyushu Dental University (ethical approval number: 18–42) and was conducted in accordance with the Declaration of Helsinki involving human participants. Written informed consent was obtained from all participants after the purpose and procedures of the study were fully elucidated.

### 2.2. Collection of Stimulated Saliva

Before the clinical assessment, stimulated saliva was collected in 15-mL sterile tubes by chewing sugar-free and odorless gum (Sheep Dental Industry Co., Wakayama, Japan) for 5 min. The saliva was subsequently centrifuged at 14,000× *g* for 10 min at 4 °C to collect only the supernatant, which was further aliquoted and immediately stored at −30 °C until analysis. The participants refrained from eating, drinking, brushing, and washing their mouth for a minimum of 2 h before saliva collection.

### 2.3. Clinical Assessments

A dentist examined the clinical periodontal probing depth (PPD), clinical attachment level (CAL), and bleeding on probing (BOP) at six sites per tooth using the periodontal probe UNC 15 (Hu-Friedy, Chicago, IL, USA) and diagnosed periodontal disease according to the clinical criteria stated in the consensus report of the 2017 World Workshop on the Classification of Periodontal and Peri-Implant Diseases and Conditions.

Additionally, the periodontal inflamed surface area (PISA) was measured in all participants and was calculated using an automatically computable EXCEL form known as the Calculate PISA probing pocket depth as an alternative to the parsprototo.info website.

### 2.4. Measurement of TNF-α, sTNF-R1, sTNF-R2, and Total Protein in the Saliva Sample

The protein levels of TNF-α, sTNF-R1, and sTNF-R2 in the saliva samples were determined according to the manufacturer’s protocols using commercially available enzyme-linked immunosorbent assay kits (R&D Systems Inc., Minneapolis, MN, USA), and the levels of total protein (TP) in the saliva samples were determined using a commercially available protein assay kit (Bio-Rad, Hercules, CA, USA).

In this study, the ratios of TNF-α, sTNF-R1, and sTNF-R2 protein levels to TP in 1 mL of saliva were expressed as TNF-α/TP, sTNF-R1/TP, and sTNF-R2/TP.

### 2.5. Statistical Analysis

The sample size was determined using the software G*Power 3.1.9.6 (Franz Faul, University of Kiel, Kiel, Germany), resulting in a minimum of 26 subjects for correlation and linear multiple regression analyses [19].

Variables between male and female participants were compared using a t-test or Mann-Whitney U test. The strength of correlations between the ratios of TNF-α/TP, sTNF-R1/TP, and sTNF-R2/TP and the clinical parameters and between the ratios of TNF-α/TP, sTNF-R1/TP, and sTNF-R2/TP were assessed using Pearson’s correlation and Spearman’s rank correlation analyses, respectively. The relationships between the ratios of TNF-α/TP, sTNF-R1/TP, and sTNF-R2/TP in saliva and between PISA and the ratios of TNFα/TP, sTNF-R1/TP, and sTNF-R2/TP in saliva were shown in the scatter plot graphs. Among the linear, quadratic, and logarithmic curve models, curve estimation by regression analysis was performed, and the quadratic curve model with the highest R-squared value and best fit was adopted as the approximation curve in each graph of the scatter plots. In addition, stepwise multiple linear regression analyses were performed using periodontal clinical parameters that were significantly correlated with TNF-α/TP, sTNF-R1/TP, or sTNF-R2/TP in Pearson’s correlation analysis as dependent variables. Each of them was analyzed using independent variables, including age, sex, number of present teeth, volume of stimulated saliva, TNF-α/TP, sTNF-R1/TP, and sTNF-R2/TP. All data analyses were performed using IBM SPSS Statistics (version 25.0; IBM Corp., Armonk, NY, USA).

## 3. Results

### 3.1. Profile of the Participants

The profiles of the participants, including sex, are shown in Table 1.

Although the mean volumes of stimulated saliva secreted for a minute (stimulated saliva) between men and women were significantly different (men (2.3 mL/min) vs. women (1.2 mL/min); *p* < 0.01, *t*-test), other clinical parameters besides saliva were not significantly different between the sexes.

### 3.2. Levels of TNF-α, sTNF-R1, sTNF-R2 Proteins, and TP in Saliva

The levels of TNF-α, sTNF-R1, sTNF-R2 proteins, and TP in 1 mL of saliva, including sex, are shown in Table 2.

The mean concentrations of TNF-α, sTNF-R1, sTNF-R2 proteins, and TP of all participants were 0.4 pg/mL (SD: 0.3), 244.1 pg/mL (165.4), 86.9 pg/mL (72.3), and 1625.5 µg/mL (768.8), respectively; there were no significant differences in the values between the sexes.

The ratios to TP of TNF-α, sTNF-R1, and sTNF-R2 in the saliva of the participants, including sex, are shown as TNF-α/TP, sTNF-R1/TP, and sTNF-R2/TP in Table 2. The TNF-α/TP, sTNF-R1/TP, and sTNF-R2/TP in all participants were 2.6 × 10^−7^ (2.3 × 10^−7^), 1.7 × 10^−4^ (1.1 × 10^−4^), and 0.6 × 10^−4^ (0.5 × 10^−4^), respectively, and all values were not significantly different between the sexes.

### 3.3. Relationships between TNF-α/TP, sTNF-R1/TP, and sTNF-R2/TP in Saliva

**Figure 1:**
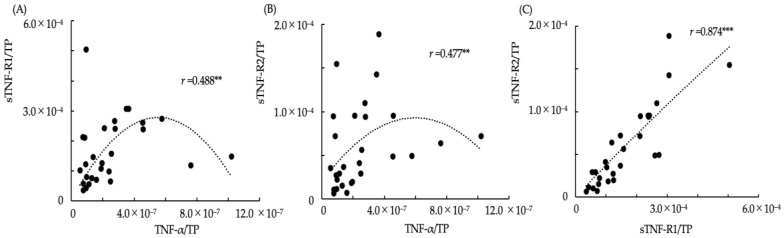
The relationships between TNF-α/TP, sTNF-R1/TP, and sTNF-R2/TP in saliva. (**A**) The relationship between TNF-α/TP and sTNF-R1/TP in saliva (*r*: Spearman’s rank coefficient, ** *p* < 0.01); (**B**) The relationship between TNF-α/TP and sTNF-R2/TP in saliva (** *p* < 0.01); (**C**) The relationship between sTNF-R1/TP and sTNF-R2/TP in saliva (*** *p* < 0.01). The dotted lines show approximation curves (quadratic curve model, (**A**) *r*^2^: 0.155; (**B**) *r*^2^: 0.155; (**C**) *r*^2^: 0.753, *p* < 0.001).

The relationships between the ratios of TNF-α/TP, sTNF-R1/TP and sTNF-R2/TP in saliva are shown in the scatter plot graphs (Figure 1). Additionally, the approximation curves determined using curve estimation by regression analysis from the scatter plots are shown in each graph. The Spearman’s rank correlation coefficients between TNF-α/TP and sTNF-R1/TP, TNF-α/TP and sTNF-R2/TP, and sTNF-R1/TP and sTNF-R2/TP were *r* = 0.488, *p* < 0.01; *r* = 0.477, *p* < 0.01; and *r* = 0.874, *p* < 0.001, respectively. In the scatter plot graph of TNF-α/TP and sTNF-R1/TP (Figure 1A) and TNF-α/TP and sTNF-R2/TP (Figure 1B), the ratios of sTNF-R1/TP and sTNF-R2/TP were observed to increase with an increase in TNF-α/TP if the ratio of TNF-α/TP was lower than approximately 4.0 × 10^−7^, whereas it decreased according to an increase in TNF-α/TP if the ratio of TNF-α/TP was more than approximately 4.0 × 10^−7^. The relationship between sTNF-R1/TP and sTNF-R2/TP was strongly correlated with interdependent changes (Figure 1C).

### 3.4. Correlations between the Ratios of TNF-α, sTNF-R1, and sTNF-R2 to TP in Saliva and Clinical Parameters of the Participants

The correlations between the ratios of TNF-α, sTNF-R1, and sTNF-R2 to TP in saliva and the clinical parameters of the participants are shown in Table 3.

There were significant correlations between TNF-α and the number of present teeth, and those of teeth with PPD > 5 mm (PPD5), and CAL > 5 mm (CAL5) (*r* = −0.487, *p* < 0.01; *r* = 0.445, *p* < 0.05; and *r* = 0.497, *p* < 0.01, respectively, Pearson’s correlation analysis), between sTNF-R1 /TP and the volume of stimulated saliva (*r* = −0.641, *p* < 0.001), and between sTNF-R2/TP and the volume of stimulated saliva, the percentage of sites with BOP to total probed sites (BOP%), and PISA (*r* = −0.598, *p* < 0.001; *r* = 0.520, *p* < 0.01; and *r* = 0.529, *p* < 0.01, respectively).

### 3.5. Stepwise Multiple Linear Regression Analysis

A stepwise multiple linear regression analysis was performed using age, sex, number of present teeth, volume of stimulated saliva, TNF-α/TP, sTNF-R1/TP, and sTNF-R2/TP as independent variables as well as BOP%, numbers of PPD5 and CAL5, and PISA, which showed significant correlations between TNF-α/TP, sTNF-R1/TP, or sTNF-R2/TP in Pearson’s correlation analysis, as the dependent variables (Table 4).

The numbers of PPD5 and CAL5 teeth showed a significant association with TNF-α and TP, respectively. BOP% indicated a significant association with sTNF-R1/TP, sTNF-R2/TP, stimulated saliva, and age, and increases in sTNF-R1/TP and stimulated saliva were associated with a reduction in BOP%. Furthermore, PISA was significantly associated with sTNF-R2/TP.

### 3.6. Relationships between PISA and the Ratios of TNFα/TP, sTNF-R1/TP, and sTNF-R2/TP in Saliva

**Figure 2:**
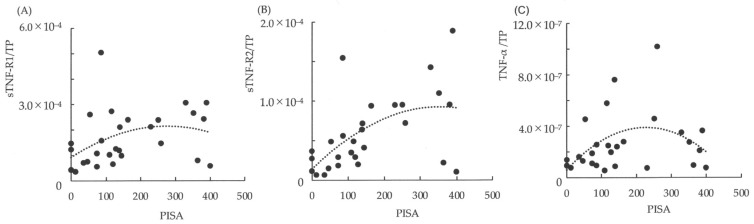
The relationships between periodontal inflamed surface area (PISA) and the ratios of TNFα/TP, sTNF-R1/TP, and sTNF-R2/TP in saliva. (**A**) The relationship between PISA and sTNF-R1/TP in saliva; (**B**) The relationship between PISA and sTNF-R2/TP in saliva; (**C**) The relationship between PISA and TNF-α/TP in saliva. Dotted lines show approximation curves (quadratic curve model, (**A**) *r*^2^: 0.131; (**B**) *r*^2^: 0.312, *p* < 0.01; (**C**) *r*^2^: 0.194).

The relationships between PISA and the ratios of TNF-α/TP, sTNF-R1/TP, and sTNF-R2/TP in saliva are shown in the scatter plot graphs (Figure 2). In addition, the approximation curve determined using curve estimation by regression analysis from the scatter plots is shown in each graph. If the PISA was < 200 mm^2^, the ratios of TNF-α/TP, sTNF-R1/TP, and sTNF-R2/TP increased linearly with the expansion of PISA. Furthermore, if the PISA exceeded 200 mm^2^, the ratio of TNF-α/TP was observed to decrease linearly with the expansion of PISA (Figure 2C), and sTNF-R1/TP did not increase with changes in PISA (Figure 2A). However, sTNF-R2/TP was observed to maintain the increase according to the expansion of PISA, although there was variation compared to the change in the level of PISA < 200 mm^2^ (Figure 2B).

## 4. Discussion

In the present study, salivary TNF-α levels were associated with an increase in the number of teeth with deep PPD and a CAL of >5 mm. TNF-α contributes to the onset of periodontal inflammation, such as periodontitis. Elevated levels of TNF-α from various cells in gingival tissue are associated with the destruction of periodontal tissues, including bone resorption [2]. Therefore, the increase in TNF-α in saliva may reflect the deterioration of periodontal tissues.

Although the dynamics of sTNF-R1 and sTNF-R2 in saliva are unknown, it was demonstrated in the present study that the mean salivary level of sTNF-R1 was higher than that of sTNF-R2. Moreover, there was a strong positive correlation between the levels of sTNF-R1 and sTNF-R2 in saliva. With regard to the dynamics between TNF-α and sTNF-R1 and sTNF-R2 in saliva, it was found in this study that both levels of sTNF-R1 and sTNF-R2 were synergistically enhanced up to a certain level of salivary TNF-α, but decreased when TNF-α exceeded a certain level. Previously, the serum levels of sTNF-R1 and sTNF-R2 in patients with ankylosing spondylitis and rheumatoid arthritis were reported to be higher than those in healthy controls [20]. Generally, sTNF-R1 and sTNF-R2 are thought to modulate and balance TNF-α activity during inflammatory events [21]. Therefore, sTNF-R1 and sTNF-R2 in saliva may be increased by oral inflammatory diseases, including periodontitis, which may act to modulate salivary TNF-α at low levels, but not at relatively high levels.

The present study is the first to depict the relationship between periodontal health and salivary levels of TNF-α, sTNF-R1, and sTNF-R2. Here, PISA was used as a parameter to quantify the amount of inflamed periodontal tissue to quantify the inflammatory burden, which indicated that salivary levels of sTNF-R2 were significantly associated with PISA in addition to BOP%, which reflects the quantitative evaluation of inflamed periodontal tissues. Although there was no significant correlation between PISA and the salivary levels of TNF-α and sTNF-R1, it seemed that increases in the levels of TNF-α, sTNF-R1, and sTNF-R2 in saliva were linked to the expansion of PISA in the early stages of inflammation, when the spread of inflamed periodontal tissue is small. Moreover, salivary TNF-α levels did not progress to a more advanced stage of inflammation, while only salivary sTNF-R2 levels tended to increase with the expansion of PISA. TNF-α was reported to upregulate the release of sTNF-R2 from human gingival fibroblasts but not that of sTNF-R1 [15]. Additionally, it was reported that the sTNF-R2/R1 ratio in GCF decreased with increasing PPD values in patients with chronic periodontitis [16], and that sTNF-R2 significantly prevented the loss of connective tissue attachment and alveolar bone in experimental periodontitis [22,23]. These findings suggest that sTNF-R2 may modulate TNF-α-mediated inflammatory responses in periodontal diseases and contribute to the prevention of aggravation, leading to periodontal tissue destruction. In the present study, the differences in the relationships between PISA and the levels of TNF-α, sTNF-R1, and sTNF-R2 in saliva may reflect the dynamics of sTNF-R1 and especially sTNF-R2. Salivary sTNF-R2 levels are significantly associated with PISA, while salivary TNF-α and sTNF-R1 levels are not, possibly because salivary sTNF-R2 continually acts to modulate salivary TNF-α in accordance with the expansion of inflamed periodontal tissue.

As a result, the reduction in salivary TNF-α levels may be induced by sTNF-R2, which may prevent development linked to the destruction of inflamed periodontal tissues. In contrast, it was evident that the salivary level of sTNF-R1 was high and mutually correlated with that of sTNF-R2, and it seemed that the salivary level of sTNF-R1 was associated with the reduction of gingival inflammation in the present study, Naturally, TNF-R1 has a high affinity against soluble TNF-α [24]. Therefore, sTNF-R1 may be associated with the initial regulation of salivary TNF-α levels with sTNF-R2 and may act as an inhibitor of gingival inflammation.

In a preliminary study, we confirmed that there were no statistical differences in the proportions of TNF-α, sTNF-R1, and sTNF-R2, which were adjusted by TP in stimulated and resting saliva, and that the collection of stimulated saliva was easier and could be obtained in larger quantities than that of resting saliva, even if saliva secretion was limited. Thus, the present study used stimulated saliva and measured the concentrations of TNF-α, sTNF-R1, and sTNF-R2 in addition to TP. Furthermore, there is a significant difference in the mean volume of stimulated saliva between the sexes, as indicated in this study, because the saliva flow rate increases with an increase in salivary gland size, and the salivary gland size differs between the sexes [25]. However, other parameters in this study, including the mean concentrations of TNF-α, sTNF-R1, sTNF-R2, and TP, seem to have no significant difference between the sexes, and not to be affected by the difference in the volume of stimulated saliva between the sexes. The origins of TNF-α, sTNF-R1, and sTNF-R2 in saliva were not clarified in this study. In our previous study, the mRNA levels of ADAM17, which is related to the production of TNF-α, sTNF-R1, and sTNF-R2, were shown to be higher in the oral buccal mucosal epithelium according to the severity of periodontal diseases [8]. Another study demonstrated that ADAM17 is strongly expressed in the epithelium of inflamed gingival tissues and regulates the generation of TNF-α in oral keratinocytes [9]. Therefore, sTNF-R1 and sTNF-R2, in addition to TNF-α, may be induced in saliva from the oral epithelium, including the buccal mucosal and gingival epithelium, which may be regulated by ADAM17 and enhanced by the development of periodontal inflammation.

Biomarkers in GCF may be more useful for assessing the state of local periodontal health compared to those in saliva; however, collecting a sufficient amount of fluid and assessing some types of biomarkers can often be difficult [26]. The present study used whole saliva to evaluate TNF-α, sTNF-R1, and sTNF-R2 levels in the oral cavity. Generally, saliva is assumed to be useful as a diagnostic fluid for oral-related diseases, including periodontitis, as it is rapid, easy, non-invasive to collect, and abundant [27]. Cytokine levels in saliva, as well as those in GCF, have been reported to correlate well with the clinical parameters of periodontal disease, suggesting that salivary cytokine levels may be more suitable for a comprehensive assessment of oral health status, including periodontal disease [17,18,26]. It is evident that TNF-α, sTNF-R1, and sTNF-R2 are included in the GCF; however, the details of these are unclear, except for the finding that the ratios of sTNF-R2/R1 in GCF significantly increase after periodontitis treatment [16]. Furthermore, the components of saliva may be affected by gingival bleeding, as the levels of TNF-α in saliva and serum are similar and are increased in patients with periodontitis [28]. Similarly, TNF-α, sTNF-R1, and sTNF-R2 in saliva are thought to reflect a variety of oral factors, such as the oral epithelium, serum, and GCF, and are influenced by periodontal health states. In particular, according to the present study, salivary sTNF-R2 levels may be used as a useful indicator to diagnose the expansion of periodontal inflammation, instead of examination using a periodontal probe.

The present study was a cross-sectional study that involved patients with slight or moderate chronic periodontitis and examined the relationships between the parameters of different periodontal health statuses of the subjects in addition to salivary TNF-α, sTNF-R1, and sTNF-R2 levels. The findings of this study are the first step in clarifying the dynamics and roles of TNF-α, sTNF-R1, and sTNF-R2 in the process of periodontal disease. In this study, data were presented separately for men and women to show the characteristics of the subjects. However, although the sample size in this study was suitable for correlation and linear multiple regression analyses, the results of the analysis obtained by comparison between the sexes may need to be considered as reference values because each sample size was small when comparing the two groups. This should be considered in the same way when comparing groups using other parameters, such as differences in age and severity of periodontal diseases in this study. Accordingly, the present study did not show other data for comparison by group, except for sex. However, the most significant limitation of this study is that we were unable to verify the same parameters in healthy subjects without periodontal disease or in patients with severe chronic periodontitis because the subjects in this study only included patients with slight or moderate chronic periodontitis. Thus, based on our findings, future studies are needed to further demonstrate the roles of sTNF-R1 and sTNF-R2 in the development of periodontal disease. Specifically, by increasing the sample size, including healthy controls and subjects with more severe periodontal health, and tracing the prognosis of the patients included in this study, it may be possible to obtain more reliable data on the status of TNF-α, sTNF-R1, and sTNF-R2 in saliva. Proinflammatory signaling activated by TNF-α is an important factor in the pathology of periodontal disease [1]. Elucidation of the dynamics of sTNF-R1 and sTNF-R2 against TNF-α in oral conditions, including saliva, is expected to contribute to the development of new procedures for the diagnosis and treatment of periodontal diseases, such as gingivitis and periodontitis.

## 5. Conclusions

The present study demonstrates for the first time that there are significant relationships between the salivary levels of TNF-α, sTNF-R1, and sTNF-R2, and that salivary sTNF-R2 is associated with the expansion of inflamed periodontal tissue.

## Figures and Tables

**Table 1:** The profiles of the participants (mean (SD)).

Parameters	All (*n* = 28)	Men (*n* = 11)	Women (*n* = 17)
Age (years)	64.7 (15.8)	68.0 (16.6)	62.6 (15.3)
Stimulated saliva (mL/min) **	1.6 (1.1)	2.3 (1.1)	1.2 (0.8)
Number of present teeth	25.2 (3.4)	25.3 (3.4)	25.1(3.5)
Mean PPD (mm)	2.5 (0.3)	2.6 (0.2)	2.4 (0.3)
Number of PPD5 teeth	1.4 (1.7)	1.0 (1.0)	1.7 (2.1)
Mean CAL (mm)	3.1 (0.5)	3.3 (0.5)	2.9 (0.5)
Number of CAL5 teeth	6.4 (5.1)	5.6 (3.8)	6.9 (5.8)
Number of BOP teeth	8.6 (6.0)	9.1 (5.2)	8.3 (6.5)
Mean BOP%	11.9 (8.7)	11.0 (9.1)	12.5 (8.7)
Mean PISA (mm^2^)	160.0 (130.7)	145.2 (136.7)	169.6 (130.0)

Stimulated saliva: mean volume of stimulated saliva, PPD: probing pocket depth, PPD5 teeth: teeth with PPD > 5 mm, CAL: clinical attachment level, CAL5 teeth: teeth with CAL > 5 mm, BOP: bleeding on probing, BOP teeth: teeth with BOP, BOP%: percentage of the number of BOP sites to total probed sites, PISA: periodontal inflamed surface area. ** *p* < 0.01, *t*-test (men vs. women).

**Table 2:** The levels of TNF-α, sTNF-RI, sTNF-R2 proteins, and total protein in the saliva of participants.

Parameters	All (*n* = 28)	Men (*n* = 11)	Women (*n* = 17)
TNF-α (pg/mL)	0.4 (0.3)	0.3 (0.1)	0.41 (0.36)
sTNF-R1 (pg/mL)	244.1 (165.4)	209.3 (126.8)	266.6 (186.3)
sTNF-R2 (pg/mL)	86.9 (72.3)	70.4 (55.8)	97.6 (81.0)
TP (µg/mL)	1625.5 (768.8)	1749.5 (589.0)	1545.3 (873.6)
TNF-α/TP	2.6 × 10^−7^ (2.3 × 10^−7^)	1.7 × 10^−7^ (1.2 × 10^−7^)	3.1 × 10^−7^ (2.6 × 10^−7^)
sTNF-R1/TP	1.7 × 10^−4^ (1.1 × 10^−4^)	1.3 × 10^−4^ (0.8 ×10^−4^)	1.9 × 10^−4^ (1.2 × 10^−4^)
sTNF-R2/TP	0.6 × 10^−4^ (0.5 × 10^−4^)	0.4 × 10^−4^ (0.2 × 10^−4^)	0.7 × 10^−4^ (0.5 ×10^−4^)

sTNF-R1: soluble tumor necrosis factor receptor type 1, sTNF-R2: soluble tumor necrosis factor receptor type 2, TNF-α: tumor necrosis factor α, TP: total protein, TNF-α/TP: ratio of TNF-α protein level to total protein in 1 mL of saliva, sTNF-R1/TP: ratio of sTNF-R1 protein level to total protein in 1 mL of saliva, sTNF-R2/TP: ratio of sTNF-R2 protein level to total protein in 1 mL of saliva.

**Table 3:** The correlations between the ratios of TNF-α, sTNF-RI, and sTNF-R2 to total protein in saliva and the clinical parameters of the participants (Pearson’s correlation coefficient).

Parameters	TNF-α/TP	sTNF-R1/TP	sTNF-R2/TP
Age (years)	−0.108	−0.181	−0.169
Stimulated saliva (mL/min)	−0.325	−0.641 ***	−0.598 ***
Number of present teeth	−0.487 **	0.186	0.051
Mean PPD (mm)	0.259	0.036	0.183
Number of PPD5 teeth	0.445 *	−0.028	0.030
Mean CAL (mm)	0.331	0.096	0.250
Number of CAL5 teeth	0.497 **	−0.002	0.272
Number of BOP teeth	−0.137	0.062	−0.039
BOP% sites	0.293	0.235	0.520 **
Mean PISA (mm^2^)	0.205	0.296	0.529 **

Values: Pearson’s correlation coefficient, * *p* < 0.05, ** *p* < 0.01, ***p < 0.001.

**Table 4:** Stepwise multiple linear regression analyses to investigate the association between the percentage of BOP sites to total sites, number of PPD > 5 mm teeth, number of CAL > 5 mm, or PISA.

Dependent Variables	Independent Variables	β	*p*	Adjusted *R*^2^
Number of PPD5 teeth ^a^	TNF-α/TP	0.445	0.018	0.167
Number of CAL5 teeth ^b^	TNF-α/TP	0.497	0.007	0.218
BOP% ^c^	sTNF-R2/TP	1.147	<0.001	0.598
	sTNF-R1/TP	−1.013	0.001	
	Stimulated saliva	−0.489	0.006	
	Age	0.275	0.037	
PISA ^d^	sTNF-R2/TP	0.529	0.004	0.252

^a^ Age, sex, volume of saliva, number of teeth, sTNF-R1/TP, and sTNF-R2/TP are not independently associated with the number of PPD5 teeth. ^b^ Age, sex, volume of saliva, number of teeth, sTNF-R1/TP, and sTNF-R2/TP are not independently associated with the number of CAL5 teeth. ^c^ Sex, number of teeth, and TNF-α/TP are not independently associated with BOP%. ^d^ Age, sex, volume of saliva, number of teeth, TNF-α/TP, and sTNF-R1/TP are not independently associated with PISA.

## Data Availability

The data presented in this study are available from the corresponding author (S.A.) upon reasonable request.
